# Integration of genomics and metabolomics for prioritization of rare disease variants: a 2018 literature review

**DOI:** 10.1007/s10545-018-0139-6

**Published:** 2018-05-02

**Authors:** Emma Graham, Jessica Lee, Magda Price, Maja Tarailo-Graovac, Allison Matthews, Udo Engelke, Jeffrey Tang, Leo A. J. Kluijtmans, Ron A. Wevers, Wyeth W. Wasserman, Clara D. M. van Karnebeek, Sara Mostafavi

**Affiliations:** 1 Department of Bioinformatics, University of British Columbia, Vancouver, BC Canada; 2 BC Children’s Hospital Research Institute, Centre for Molecular Medicine and Therapeutics, University of British Columbia, Vancouver, BC Canada; 3 Department of Medical Genetics, University of British Columbia, Vancouver, BC Canada; 4 Department of Biochemistry and Molecular Biology, Cumming School of Medicine, University of Calgary, Calgary, AB Canada; 5 Department of Medical Genetics, Cumming School of Medicine, University of Calgary, Calgary, AB Canada; 6 Alberta Children’s Hospital Research Institute, University of Calgary, Calgary, AB Canada; 7 Department of Pediatrics, BC Children’s Hospital Research Institute, Vancouver, BC Canada; 8 Translational Metabolic Laboratory, Department of Laboratory Medicine, Radboud University Medical Center, Nijmegen, the Netherlands; 9 Departments of Pediatrics and Clinical Genetics, Emma Children’s Hospital, Academic Medical Centre, Amsterdam, The Netherlands; 10 Department of Statistics, University of British Columbia, Vancouver, BC Canada

**Keywords:** Metabolomics, Genomics, Omic integration, Inborn errors of metabolism, Variant prioritization

## Abstract

Many inborn errors of metabolism (IEMs) are amenable to treatment; therefore, early diagnosis and treatment is imperative. Despite recent advances, the genetic basis of many metabolic phenotypes remains unknown. For discovery purposes, whole exome sequencing (WES) variant prioritization coupled with clinical and bioinformatics expertise is the primary method used to identify novel disease-causing variants; however, causation is often difficult to establish due to the number of plausible variants. Integrated analysis of untargeted metabolomics (UM) and WES or whole genome sequencing (WGS) data is a promising systematic approach for identifying disease-causing variants. In this review, we provide a literature-based overview of UM methods utilizing liquid chromatography mass spectrometry (LC-MS), and assess approaches to integrating WES/WGS and LC-MS UM data for the discovery and prioritization of variants causing IEMs. To embed this integrated -omics approach in the clinic, expansion of gene-metabolite annotations and metabolomic feature-to-metabolite mapping methods are needed.

## Introduction

Inborn errors of metabolism (IEMs) are the largest group of genetic diseases amenable to causal therapy, and are caused by genetic variants that disrupt the function of enzymes or other proteins involved in cellular metabolism, leading to energy deficit and/or accumulation of toxins (Van Bokhoven 2011; del Rosario et al 2012; Rauch et al 2012; Ellison et al 2013). Early diagnosis, enabled by newborn metabolic screening programs and genetics profiling, is pivotal so that treatment can be initiated before the onset of irreversible progressive damage to the central nervous system, which in some cases can result in intellectual disability disorder (IDD) and damage to additional organ systems.

There are currently more than 100 treatable IEMs, but for many phenotypes the genetic basis remains to be discovered (Van Karnebeek and Stockler 2012). Cases for which the causal gene was recently identified have in turn provided insights and opportunities for interventions targeting their downstream molecular or cellular abnormalities (Collins et al 2010; Horvath et al 2016; Karnebeek et al 2016). These efforts have been cataloged in the online resource IEMbase, which provides further information on the etiologies and treatment of over 500 IEM disorders (Blau et al 2014).

Whole exome sequencing (WES) is the primary tool for discovery of the genetic basis of IEMs, and thus establishment of a genetic-based diagnosis that, in some cases, can lead to improved outcomes through targeted interventions. The promise of this approach was illustrated by a recent neurometabolic gene discovery study (Tarailo-Graovac et al 2016), in which deep phenotyping and WES achieved a diagnostic yield of 68% in patients with unexplained phenotypes, identified novel human disease genes, and most importantly enabled targeted intervention for improved outcomes in 44% of the patients. Overall, published studies applying WES coupled with variant prioritization in patients with unexplained phenotypes are successful in identifying the underlying cause in 16 to 68% of patients (Tarailo-Graovac et al 2016).

However, with our current limited knowledge of variant pathogenicity and the impact of rare variants, variant prioritization algorithms that aim to completely automate the process of prioritization fail to identify the causal variant in a substantial number of patients. Further, variants that are identified as plausible often have a low level of supporting evidence, and are thus not adequate to establish a genetic-based diagnosis. Using multiple types of personalized “-omic” data is a promising approach to address the evidence gap in support of an IEM diagnosis. The integration of metabolomics data with WES/WGS data to identify genes causing IEM is a prime example of this approach. For example, a diagnosis of maple syrup urine disease can be supported by 1) pathogenic variants in either *DBT*, *BCKDHB* or *BCKDHA*, 2) high levels of amino acids, such as allo-isoleucine, isoleucine, leucine, and valine, and 3) branched-chain oxoacids (Strauss et al 2013). These biochemical biomarkers can be detected individually (targeted metabolomics), or as part of a broader characterization of the metabolome (untargeted metabolomics). Recently, the unbiased approach afforded by untargeted metabolomics has increased in popularity due to decreasing costs, lack of required parameter tuning, and opportunities for pathway analysis (Johnson et al 2016). In this literature review, we provide an overview (anno 2018) of WES-enabled variant prioritization, untargeted metabolomics methods utilizing liquid chromatography MS (LC-MS), and assess approaches to integrating WES/WGS and LC-MS untargeted metabolomics data for the discovery and prioritization of variants causing IEMs.

### Overview of WES-enabled prioritization of causative variants

Bioinformatic-driven variant prioritization involves multiple filtering steps that incorporate prior knowledge about allele population frequency and predicted pathogenicity. Databases, such as ExAC, dbSNP, and gnomAD, provide information about allele frequencies seen in the general population, which are then used to filter out common and likely non-pathogenic variants in the patient (Smigielski et al 2000; Exome Aggregate Consortium 2016). Once identified as pathogenic through use of in silico prediction tools (such as PolyPhen-2 and SIFT), genomic data from the individual’s parents is then used to filter variants according to Mendelian models of inheritance, making the parents the individual’s “controls”(Ng and Henikoff 2003; Adzhubei et al 2013). These different types of controls allow for the isolation of pathogenic variants, and the assignment of mode of inheritance. However, it should be noted that some studies have questioned whether genomic databases may in fact contain individuals with disease-associated genotypes but no clinical presentation of the underlying disease at the time of the inclusion, as more than 2.8% of the ExAC population was found to carry likely/pathogenic genotypes reported in ClinVar, (Tarailo-Graovac et al 2017). Continued efforts to combine clinical and genetic data will play an important role in clarifying the pathogenicity and frequency of variants in genetic backgrounds.

**Fig. 1:**
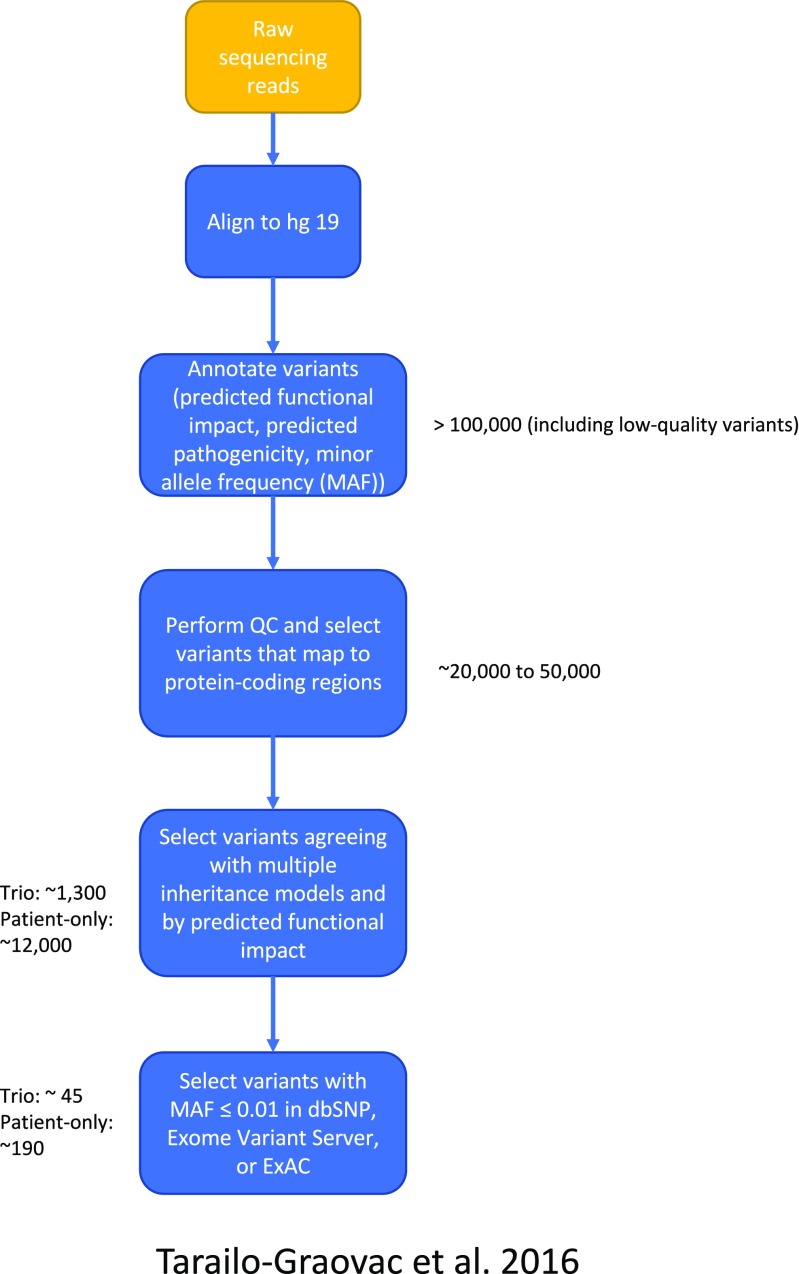
WES rare variant analysis pipeline for the detection of inborn errors of metabolism causing neurometabolic disorders, as used in Tarailo-Graovac et al 2016. Given raw sequencing reads for each patient, this pipeline identifies a conservative list of candidate variants (MAF ≤ 0.01). First, raw reads (FASTQ files) are aligned to the human genome (hg19 or equivalent). Second, variants are annotated using published software programs like ANNOVAR. Third, variants that do not map to protein-coding regions, or that do not pass QC steps are removed. Fourth, variants that do not agree with multiple inheritance models and that would not agree with the observed phenotypic effect are removed. Finally, rare variants are selected by removing variants with annotated minor allele frequencies (MAF) greater than 0.01

A sample WES variant filtering pipeline used in Tarailo-Graovac et al 2016 is detailed in Fig. 1. In the case of WES, in which around 20,000 variants are observed in protein coding regions per individual, standard filtering steps typically enable researchers to reduce the number of variants to an order of 10 to 100 candidate variants depending on the WES study design (e.g., access to trio data and pedigree structure) (Yang et al 2013; Belkadi et al 2015). For the challenging task of identifying the needle in the haystack, i.e., the single causative variant, clinical input and extensive discussion among physicians, genetic counselors, and bioinformaticians is typically needed; for genes previously unreported to cause human disease, identification of other families with similar phenotypes and other variants in the same gene as well as in vitro functional studies are required as evidence for validation of etiology (Tarailo-Graovac et al 2016). However, for a substantial number of cases (e.g., ~30–40%), the number of potential candidate variants often result in long processing times and inherent uncertainty in causation; especially for variants previously unreported in human disease, of poor sequencing quality or unknown significance (Bertier et al 2016). As we discuss later, multi-omic data integration offers a promising approach to help address this challenge.

### Methods for acquisition and analysis of untargeted metabolomics data

**Fig. 2:**
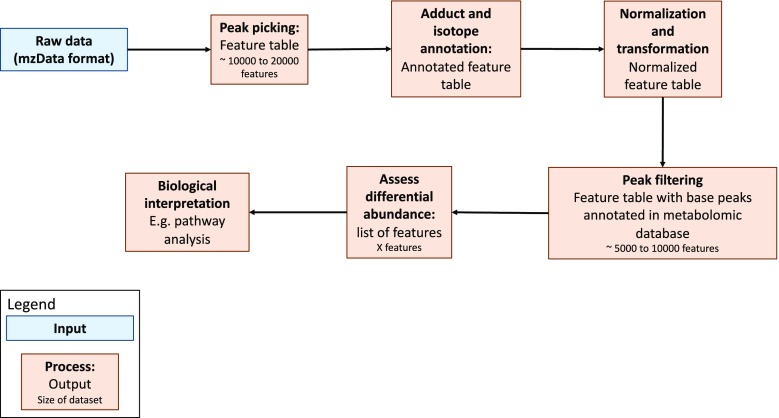
Sample LC-MS metabolomics analysis pipeline. Briefly, raw metabolomics data can be processed using freely available processing software (e.g., XCMS), annotated (e.g., CAMERA), normalized (e.g., through use of internal standards), and filtered. Differentially abundant metabolites can be isolated using univariate or multivariate tests. Biological interpretation such as pathway analysis can be performed using published metabolomic databases (e.g., HMDB, BioCyc, METLIN)

Since IEMs result from a malfunction of protein-coding genes, many of which control the concentration of a variety of metabolites, biochemical tests of known IEM-related metabolites have long been performed for IEM diagnosis. The simultaneous assay of many IEM biomarkers through the use of untargeted metabolomics is an active research area. In this section, we first provide an overview of existing approaches for processing and analyzing *untargeted* LC-MS metabolomics data for IEM diagnosis and discovery. This includes three critical components: 1) data representation and normalization, 2) identification of significant variables (“features”), and 3) association of variables/features with *known* metabolites. An overview of a hypothetical untargeted LC-MS pipeline is provided in Fig. 2.

#### Acquiring untargeted metabolomic data

In general, metabolomics quantifies a subset of small molecules (metabolites) in a tissue or body fluid using either nuclear magnetic resonance (NMR) spectroscopy or mass spectrometry (MS) (Johnson et al 2016). NMR spectroscopy quantifies solution-state molecular structures based on atom-centered nuclear interactions. NMR spectroscopy is inexpensive, capable of high throughput analysis, and highly reproducible; however, it lacks sensitivity and is generally only able to quantify metabolites of medium to high abundance. For this reason, MS based quantification has primarily been used in the context of IEM diagnosis and discovery.

In mass-spectrometry (MS) based quantification, metabolites are first chromatographically separated and quantified in a semi-quantitative manner using high resolution mass spectrometers in detection modes that measure both positive and negatively charged ions produced through electrospray ionization (ESI). MS separation techniques include liquid chromatography, capillary electrophoresis, gas chromatography, and ultra-performance liquid chromatography (Zhang et al 2012). No single chromatographic separation protocol can quantify all metabolites in a sample. Therefore, to completely capture all metabolites, multiple chromatographic methods must be used. For example, reverse-phase LC quantifies non-polar to slightly polar molecules, while hydrophilic interaction LC detects strongly polar to slightly polar molecules (Bajad et al 2006; Roberts et al 2012). This review will focus on liquid chromatography coupled MS (LC-MS), as it quantifies the widest range of metabolite polarity, and is widely used. When coupled with LC, the most common means of separation are reverse phase liquid chromatography (RPLC) for separation of hydrophobic metabolites, and hydrophilic interaction chromatography (HILIC) for the separation of hydrophilic metabolites (Zhou and Yin 2016). MS platforms commonly used for untargeted metabolomics studies include low resolution techniques, such as triple quadrupole (QQQ), quadrupole-ion trap (QIT), and high resolution techniques, such as quadrupole-time of flight (Q-TOF), quadrupole Orbitrap (Q-Orbitrap) and Fourier transform ion cyclotron resonance mass spectrometry (FTICR-MS).

#### Processing LC-MS data

**Fig. 3:**
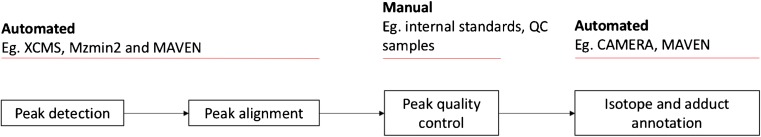
Untargeted metabolomics pre-processing pipeline. A combination of automated and manual steps are used to prepare metabolomics data for downstream analysis. The algorithms listed are only examples of tools that could be used in each step

An overview of the manual and automatic components of the LC-MS pre-processing pipeline is detailed in Fig. 3. The first step is to convert an LC-MS-produced dataset for a single individual into a list of “features” (defined as the combination of mass to charge (m/z) ratio and retention time) and their intensities. A variety of software packages designed to process metabolomic data have been developed for this purpose, of which XCMS, Mzmine2, and MAVEN are among the most popular (Katajamaa et al 2006; Tautenhahn et al 2008; Melamud et al 2010). Each pipeline involves three steps: 1) “peak selection”, in which features are identified and quantified, 2) retention time alignment, whereby intensity profiles of consecutive samples are aligned to allow maximal feature overlap, and 3) adduct and isotope annotation. The most prominent difference between existing packages involves their approach toward assessing peak quality during the peak selection step. Both XCMS and Mzmine2 define low quality peaks according to a user-defined signal-to-noise ratio cutoff threshold; in contrast, MAVEN uses a machine-learning (neural network) approach. Because an independent comparison of MAVEN, Mzmine2, and XCMS has not yet been completed, one recommendation is to analyze metabolomics data using several packages and remove peaks that are not robustly identified by multiple algorithms (Tautenhahn et al 2008). This is one of several methods that aims to minimize false positives, as it has been shown that up to 90% of features in an LC-MS experiment are non-biological noise or degenerate in a typical LC-MS experiment (Mahieu and Patti 2017). Other methods include curating databases of confirmed features identified using different separation techniques, and removing features not profiled in the corresponding database (Mahieu and Patti 2017). An additional approach is to confirm the presence of the feature in a technical replicate. In practice, it is difficult to identify the same metabolites across replicates, as retention times may differ, and is therefore most often done in targeted metabolomics, in which only a small subset of features are quantified (Crews et al 2009). In addition to the above, another method for identifying robust features involves removing features that are not detected in a set of quality control (QC) samples consisting of either a set number of defined metabolites, or a combination of all tested samples (pooled sample) (Brodsky et al 2010; Godzien et al 2015).

#### Normalizing LC-MS data

To be biologically informative, raw intensities need to be corrected for a) batch effects, b) missing values, and c) inter-sample variation. This section describes standard approaches used for such normalizations.

As a first step, raw intensities of each feature produced from data processing packages typically need to be corrected for systematic variation due to batch effects. In metabolomics data, a common type of batch effect is “chemical drift”. This drift—caused by changes in signal that occur as metabolites interact with each other while waiting to be analyzed—can be corrected if QC samples are analyzed in between experimental samples (Vaikenborg et al 2009; Shen et al 2016). While these corrections are not always performed, they have been shown to minimize inter-batch variation (Alonso-herranz et al 2015). Dimensionality reduction approaches such as PCA allow for visualizing systematic trends in high-dimensional data, and thus are powerful tools for assessing batch effects.

Missing values can result from a variety of processes, and thus require a nuanced approach. Specifically, a missing value, which is an intensity of zero or infinity, can be created from a metabolite existing in one sample but 1) not existing in another, 2) existing at a concentration below an instrument’s limit of detection or 3) existing at a concentration above an instrument’s limit of detection. The problem of missing values can best be improved by increasing the sensitivity of detection of the MS platform. Numerous strategies have been developed to reduce missing values through a group of analytic techniques called missing value imputation (MVI). The utility of these techniques has empirically been found to depend on whether univariate or multivariate techniques are used to detect differentially abundant features (Karpievitch et al 2012).

Subsequently to above, both sample-wise and feature-wise normalization methods that concurrently consider multiple samples, are typically applied to adjust for technical and biological variation. Sample-wise normalization methods involve constructing scaling factors for each sample, and include quantile, linear baseline, total ion count (TIC), and LOESS normalization (Wu and Li 2016). These methods adjust for technical factors that may have affected the entire sample. Feature-wise normalization methods involve constructing scaling factors for each feature, and include centering, scaling, and transformations (Bolstad et al 2003; van den Berg et al 2006). These approaches minimize the intensity differences between metabolites with low or high abundance, allowing relative perturbations of each metabolite to be compared. Usually, both sample-wise and feature-wise normalization methods are applied during pre-processing; however, the type of each is data-set dependent, as no gold standard approach exists.

#### Testing for significant features in IEM studies

In a typical experimental design relevant to IEMs, one typically measures metabolomics data for a set of patients only or a set of patients and some controls (i.e., case/control design). Because each case is likely unique (i.e., may represent a unique disease caused by a rare genetic variant), data is usually analyzed for one patient at a time and compared against a) controls or b) other patients. Both parametric (e.g., t-test, ANOVA) and non-parametric (e.g., Mann-Whitney U-test, Wilcoxon-signed rank and Kruskal-Wallis) tests can be used to identify differentially abundant features in a given patient sample. When pursuing parametric tests, which typically have more statistical power compared to non-parametric tests, care must be taken to transform data so that it is distributed according to the expectation of the test (e.g., Gaussian for t-test). Correction for multiple testing must also be performed in a way that balances the generation of false positives and false negatives. In contrast to studies of common disease, with this type of analysis, one seeks to identify “outlier” features, as they highlight abnormal metabolites that may be pathogenic. Thus, availability of biological and technical replicates is important in confirming that a given metabolite value is a “biological” outlier, rather than an artifact of technical variation.

In metabolomic studies, selection of “control” samples (or comparators) that are as similar as possible to the patient being studied is paramount to reducing noise. This is difficult due to the numerous factors that influence the metabolome (i.e., age, sex, ethnicity, food consumption, and time of day). Selection of controls often depends on patient availability, and the type of bio-fluid analyzed; finding suitable controls is much easier for analysis of urine samples, and much more difficult for plasma and CSF samples, due to the relative ease at which these samples can be provided. Because the metabolome has been estimated to have a median heritability of approximately 50%, the trio structure has been suggested as a possible replacement for the classical case-control design, as it may enable the removal of metabolomic features attributable to non-disease related heritable phenotypes, allowing researchers to isolate features related to a specific neurometabolic phenotype. When IEMs are predominantly recessive, parents would likely show an abnormal profile for metabolites related to a heterozygous gene variant, which due to the effects of a suspected knock-down, would be magnified in the bi-allelic patient (Long et al 2017). However, due to inherent uncertainty in quantification, the significant impact of age, gender and diet, and varying heritability of each metabolite, the human metabolome needs to be explored further before the trio structure can be robustly used in this manner. Overall, like any other omics study of dynamic molecular traits, experimental designs that enable robust statistical adjustments for the effect of demographical and environmental factors are of key importance for identifying meaningful disease-associated metabolites. At the least, care should be taken to utilize metabolomic controls that share as many characteristics as possible with the population being studied.

#### Annotating features: adducts, isotopes, and metabolites

Once features have been identified, they can be annotated as an adduct or isotope of a particular metabolite. An adduct is an ionized metabolite that has become associated with another ion through electrospray ionization (ESI), most commonly H^+^, Na^+^, K^+^, and H_2_0. An isotope is a metabolite that is composed of elements that are not in their most abundant form. A metabolite’s most abundant isotopic form generally corresponds to its most abundant features. The most readily quantifiable adduct depends on the chromatographic separation performed (Keller et al 2008). Annotation of isotopes and adducts corresponding to a particular metabolite reduces the multiple testing burden by enabling the removal of features belonging to the same metabolite. Removal of redundant features is performed at the discretion of the researcher, as no standard filtering approach exists. In Mzmine2 and MAVEN packages, adduct and isotope annotation is performed automatically, whereas if XCMS is utilized, an external package (such as CAMERA) must be used to make these annotations (Kuhl et al 2012).

The putative metabolite mass annotated in the peak-annotation step described earlier is used to map a specific feature to known metabolite(s). Databases that include mass, adduct, spectra, and structure data are then used to match metabolite masses to known metabolites that fall within the specific mass accuracy of the mass spectrometer used. Several such databases exist, such as the Human Metabolome Database (HMDB), Recon2, BioCyc, and METLIN (Petri and Schmidt-Dannert 2004; Smith et al 2005; Wishart et al 2007; Thiele et al 2013). The HMDB in particular contains information on endogenous, food-based and drug-related metabolites found in urine, CSF, and plasma of humans. The fact that only metabolites found in humans are profiled makes this database particularly useful for mapping features identified through untargeted metabolomic methods, as the entire database can be utilized without a priori knowledge of each metabolite’s origin. At the time of writing of this manuscript, it contains over 114,100 metabolites annotated with structure and chemical properties, a portion of which are also associated with specific genes (*n* = 5701). Of these metabolites, 19.5% have been detected in a biofluid, and 81.5% are predicted or expected. Its sister database, the Small Molecule Pathway Database (SMPDB), annotates a portion of genes and metabolites to specific small molecule pathways. Together, the HMDB and SMPDB facilitate biological interpretation at the gene and pathway level. Limitations of these databases include the relatively small number of detected and quantified metabolites, as well as the relevant paucity of genes annotated to both HMDB and SMPDB.

Confirming the “true” identity of a specific feature is challenging because each neutral mass can be annotated to multiple isobaric metabolites, (i.e., one-to-many mapping). Narrowing down the identity of a given feature is currently an active area of research (Li et al 2013; Pirhaji et al 2016). Public databases that contain metabolite masses and MS/MS spectra can assist in confirming metabolite identities, in cases where mass spectra are available (Wishart et al 2007). Additionally, “internal standards”, or radiolabeled compounds that can be easily identified through isotopic analysis, have been used to aid feature identification in targeted metabolomics as well as untargeted lipidomics (detection of all lipids in the metabolome), as they allow researchers to benchmark when certain ions elute over time (i.e., their retention time) (Sysi-Aho et al 2007; Ejigu et al 2013; Weindl et al 2015). Validation of the mapping between a feature and its assigned metabolite can be achieved by analyzing a purchased chemical standard through identical processing techniques, and comparing its m/z ratio, potential ion-source fragments, and retention time to that of an experimentally-derived feature.

#### Identifying IEMs through untargeted metabolomic analysis

The creation of processing tools and metabolomic databases has greatly facilitated the use of untargeted metabolomics in diagnosing IEMs. Both univariate and multivariate tests have been used to identify biomarkers of IEMs through untargeted metabolomics (Wikoff et al 2007; Dercksen et al 2013; Venter et al 2014; Najdekr et al 2015; Abella et al, 2016; Kennedy et al 2017; Pappan et al 2017). Many of these identified biomarkers have been added to newborn IEM screenings, enabling the detection of a wider variety of IEMs. Recently, untargeted metabolomics demonstrated its utility as a replacement for traditional newborn dry blood spot screenings, as it was successfully used to identify 20 of 21 IEMs (with each IEM represented by more than two patients) (Miller et al 2015). Challenges with this method include separating noise (unrelated food and environmental influences) from disease signal and identifying isobaric compounds. Because of these challenges, untargeted metabolomics alone is unlikely to usurp traditional genomics-based methods that identify causative genes for novel IEMs. However, cross-omic studies that integrate genomics and metabolomics have been initiated for this purpose. The benefits and drawbacks of integrating genetic and metabolomic data for the purpose of identifying both known and unknown IEMs are addressed in the subsequent section.

#### Integrating genomics and metabolomics

Development of models and algorithms for integrated analysis of genomics and metabolomics data is an active area of research. To perform an up-to-date review of published articles pertaining to the integration of genomics and metabolomics data, the Pubmed NCBI database and Google Scholar databases were queried for all articles published between Jan 1966 and July 2017 that contained the phrases “metabolomics” and “whole exome sequencing”, “metabolomics” and “whole genome sequencing” or “metabolomics” and “genomics”. Conference abstracts as well as articles utilizing targeted metabolomics were excluded, leaving a total of 17 articles for review.

Integration of genomic and metabolomic data has been performed for two primary purposes: to identify 1) metabolically active loci and 2) genes relevant to a disease phenotype. Most existing methods for combining genomic and metabolomic data conceptually follow from the former purpose. These studies aim to increase our understanding of genetically determined metabolic phenotypes at the population level. Specifically, population-based studies have combined genotyping microarray data (Gieger et al 2008; Hicks et al 2009; Illig et al 2010; Suhre et al 2011; Demirkan et al 2012; Tukiainen et al 2012; Kettunen et al 2012; Shin et al 2014; Draisma et al 2015; Rhee et al 2016; Long et al 2017) or WES/WGS data (Guo et al 2015; Yazdani et al 2016; Yu et al 2016) with metabolomics data, using quantitative trait loci (QTL) analysis, to metabolically identify loci and characterize the impact of common genetic variation (also known as heritability) on metabolite abundance. In so-called metabolite QTL (mQTL) analysis, linear regression is used to associate genetic variants with individual metabolite intensities (or metabolite ratios) for 2000 to 8000 subjects, allowing for the identification of metabolites associated with genetic loci. To reduce spurious associations, most studies restrict their analysis to common variants (e.g., MAF ≥ 5%) in addition to restricting the analysis to pairs of variants and metabolic loci that are located near each other (i.e., “cis” association analysis) (Tukiainen et al 2012). Such mQTL analyses have identified numerous disease biomarkers by associating variants in known disease-causing genes with metabolites. In all studies examined, loci strongly associated with metabolite intensities or ratios—termed “metabotypes”—have predominantly been found to map close to or in genes associated with enzymes, transporters, and regulators of metabolism, facilitating biological interpretation. Associations between variants in genes of unknown function and metabolites are more difficult to interpret, and have required additional experimental investigation.

Consistent with transcriptomic-based QTL studies (e.g., eQTL studies), it has been reported that, on average, genetic variation is a stronger predictor of metabolite variance across individuals, compared to demographic and symptom-based clinical covariates (Rhee et al 2013; Shin et al 2014). Heritability estimates have varied across classes of metabolites. Shin et al found the heritability of amino acids (e.g., carnosine, h^2^ = 0.86, *P* = 6.8 × 10^−4^) to be higher than lipids (e.g., lysophosphatidylcholine, h^2^ = 0.46, *P* = 2.0 × 10^−7^), and that of essential amino acids (mean h^2^ = 0.29) to be lower than non-essential amino acids (mean h^2^ = 0.53), suggesting that some metabolites are more influenced by genomic variation than others, as one might expect (Shin et al 2014).

Rare variants (0.5% ≤ minor allele frequency (MAF) ≥ 5%) have been found to have a larger effect size than common variants (MAF > 5%) (Long et al 2017). However, because *association analysis (*e.g.*, mQTL* analysis) is statistically challenging and underpowered in the rare disease setting (e.g., when a variant has only been observed once), the effects of rare variants have primarily been studied manually by considering their predicted effects. Long et al identified the effect of 17 rare coding variants (SnpEff annotations, such as “stop”, “missense” or “frame”) by first manually identifying an outlier metabolite that based on biological plausibility could be affected by the variant, and then confirming the presence of this putative rare variant and outlier metabolite combination in at least one other sample (Long et al 2017). Guo et al examined the effect of rare coding variants by assessing the overlap of genes in perturbed metabolic pathways (i.e biochemical pathways with at least one outlier metabolite) with rare exon variants (Guo et al 2015). These studies indeed show that rare variants can have a large effect on metabolic variation, but the small number of rare variant-metabolite relationships yet identified suggest that clarifying their role in a systematic manner will likely require a more nuanced approach.

Studies aiming to explore rare disease using smaller sample sizes have used metabolomics in conjunction with curated biochemical knowledge for the second purpose (mentioned above): deriving disease-specific biological insights. In a “pathway based approach”, genes in enriched metabolic pathways were found to harbor variants that explained the patient’s biochemical phenotype (Guo et al 2015). Several studies have also reported untargeted metabolomics’ utility in quantifying gene-associated metabolites to provide evidence a variant is disease-causing (Gauba et al 2012; Abella et al, 2016; Pappan et al 2017). An example of this approach is our group’s recent study, which used untargeted metabolomics to demonstrate that a bi-allelic variant in *N*-acetylneuraminic acid synthase (*NANS*) in patients with infantile-onset severe developmental delay and skeletal dysplasia was reflected in high levels of N-acetylneuraminic acid (Van Karnebeek et al, 2016). Confirmation of high levels of this enzymatic substrate of *NANS* suggested that the clinical phenotype was likely caused by an enzymatic deficiency in *NANS*. Normalization of skeletal dysplasia in a zebrafish model with knocked-out *nansa and nansb* (zebrafish orthologs for human *NANS*) occurred after supplementation with sialic acid, shedding light on a possible treatment. These findings support the idea that metabolomics and genomics (i.e., mircorarrays/WES/WGS) can synergize and an integrated approach can be used to facilitate variant filtering and improve diagnosis and IEM discovery. As of yet, genome-wide integration has mainly been performed for exploratory purposes. Challenges facing the integration of these two -omic technologies must be addressed before use in clinical diagnostics.

## Major challenges to the integration of genomics and LC-MS metabolomics

Challenges to streamlining the multi-omic approach for IEMs exist. These can broadly be divided into those concerning the technical aspects of metabolite quantification and identification, and those concerning the biological interpretation of results. On the technical side, it is currently impossible to know the number of unique metabolites in the typical plasma/CSF/urine metabolome, as no LC-MS protocol is capable of identifying all metabolites. This means that for experiments aiming to capture an unbiased snapshot of the metabolome, a combination of chromatography techniques must be used. Comparisons across platforms are difficult to make, as little is known about how results from different analytic techniques can be compared, although some efforts have been made (Büscher et al 2009; Yet et al 2016; Leuthold et al 2017). Further, only approximately 65% of metabolites are quantifiable in all three body fluids (plasma, urine, and CSF), indicating that care must also be taken to select the relevant biofluid (Kennedy et al 2017). Additionally, different pre-processing algorithms may have a large effect on feature detection and adduct annotation. This renders analysis reproducibility difficult. On the biological interpretation side, there is a lack of established methods for mapping a genomic perturbation to its downstream (directly and indirectly) impacted metabolites in the rare disease context. mQTL studies are underpowered, particularly for those caused by rare variants, making it difficult for small studies to form novel IEM insights. Finally, diagnostic analyses are limited by incomplete annotation of gene-metabolite associations in databases such as the HMDB (at time of writing, only 5701 genes are associated with metabolites in the HMDB).

The challenges facing use of metabolomics in IEM diagnositics are best illustrated through the exploration of four cases reported in our TIDEX neurometabolic gene discovery study (Tarailo-Graovac et al, 2016), each with a known IEM-causing variant: a *CPT1A* variant, *NANS* variant, *DYRK1A* variant, and *SCN2A* variant, respectively (Table 1). *CPT1A* (carnitine O-palmitoyltransferase 1, liver isoform) and *NANS* (N-acetylneuraminate synthase) are enzymes that catalyze highly specific interactions, and do not share many metabolites with other genes. In contrast, *SCN2A (*sodium channel protein type 2 subunit alpha*)*, a transmembrane sodium ion transporter, interacts with the common metabolites ATP, sodium and water, and *DYRK1A* (dual specificity tyrosine-phosphorylation-regulated kinase 1A), a phosphotransferase, also interacts with ATP and ADP. The metabolites associated with *SCN2A* and *DYRK1A* would be less likely to be identified as differentially abundant, as ATP and ADP are used in multiple metabolic pathways and are under strong homeostatic control. This is echoed by Nicholson et al, who notes that unlike in eQTL studies, there is not a one-to-one mapping between a metabolite and a gene (Nicholson et al 2011). Because more statistical tests are performed in mQTL studies, effect sizes must be larger to reach statistical significance. This suggests that even when a robust snapshot of the metabolome is procured using multiple metabolomic techniques, metabolomics may only be useful in confirming perturbations/validating deleterious impact in genes that interact with metabolites under weak homeostatic control, as they are likely to have larger effect sizes. Metabolomics would, therefore, not be of use in the prioritization of *SCN2A* and *DYRK1A*. Future work is needed to address these challenges, and to formally understand in which situations metabolomics is of most value.

**Table 1:** Identified IEM genes, their functions, and number of associated metabolites (as listed in the Human Metabolome Database)

Gene	Function	Number of annotated metabolites
*CPT1A* (carnitine O-palmitoyltransferase 1, liver isoform)	Catalyzes the transfer of the acyl group of long-chain fatty acid-CoA conjugates onto carnitine, an essential step for the mitochondrial uptake of long-chain fatty acids and their subsequent beta-oxidation in the mitochondrion	13,757
*NANS* (N-acetylneuraminate synthase)	Produces phosphorylated and unphosphorylated forms of N-acetylneuraminic acid (Neu5Ac) and 2-keto-3-deoxy-D-glycero-D-galacto-nononic acid (KDN)	9
*DYRK1A* (dual specificity tyrosine-phosphorylation-regulated kinase 1A)	Phosphotransferase	2
*SCN2A* (sodium channel protein type 2 subunit alpha)	Sodium ion membrane transporter	5

## Future directions

In order for successful integration of genomics and LC-MS based metabolomics in the clinical diagnostics of IEMs, several areas of improvement must be addressed. First, existing feature detection and adduct/isotope annotation methods must be refined and benchmarked for use in clinical metabolomics. Several publicly available databases with known chemical compositions have been generated for this purpose (Kenar et al 2014). Second, exploration of gene-metabolite associations through mQTL studies followed by biochemical validation is needed to expand annotations in databases such as the HMDB. Due to the magnitude of this task, and relevance to other fields, a large-scale collaboration between geneticists and clinical chemists aiming to map the human genome onto the human metabolome may prove worthwhile. This type of effort may also elucidate which genetic perturbations are most easily detectable through metabolomic analysis; thereby, clarifying innate biases in integrated omics analyses. Third, additional computational methods that integrate genetics and metabolomics data must be explored. Toward this end, network models that take into account the interactions between genes and metabolites have been used to identify metabolic pathways perturbed in disease (Krumsiek et al 2012; Li et al 2013; Pirhaji et al 2016). So far, these methods have primarily been used to identify signatures in relatively common diseases. Building integrated genomic and metabolomic networks in the rare disease context may prove difficult; therefore, further research is needed.
